# The Latest FDA-Approved Pharmaceuticals Containing Fragments of Tailor-Made Amino Acids and Fluorine

**DOI:** 10.3390/ph15080999

**Published:** 2022-08-14

**Authors:** Qian Wang, Jianlin Han, Alexander Sorochinsky, Aitor Landa, Greg Butler, Vadim A. Soloshonok

**Affiliations:** 1 Jiangsu Co-Innovation Center of Efficient Processing and Utilization of Forest Resources, College of Chemical Engineering, Nanjing Forestry University, Nanjing 210037, China; 2 V.P. Kukhar Institute of Bioorganic Chemistry and Petrochemistry, The National Academy of Sciences of Ukraine, 1 Murmanska Str., 02094 Kyiv, Ukraine; 3 Department of Organic Chemistry I, Faculty of Chemistry, University of the Basque Country UPV/EHU, Paseo Manuel Lardizábal 3, 20018 San Sebastián, Spain; 4 Oakwood Chemical, Inc., 730 Columbia Hwy. N, Estill, SC 29918, USA; 5 IKERBASQUE, Basque Foundation for Science, Alameda Urquijo 36-5, Plaza Bizkaia, 48011 Bilbao, Spain

**Keywords:** pharmaceuticals, tailor-made amino acids, fluorine, drug design, asymmetric synthesis

## Abstract

Nowadays, the selective introduction of fluorine into bioactive compounds is a mature strategy in the design of drugs allowing to increase efficiency, biological half-life and bio-absorption. On the other hand, amino acids (AAs) represent one of the most ubiquitious classes of naturally occurring organic compounds, which are found in over 40% of newly marked small-molecule pharmaceutical drugs and medical formulations. The primary goal of this work is to underscore two major trends in the design of modern pharmaceuticals. The first is dealing with the unique structural characteristics provided by the structure of amino acids featuring an abundance of functionality and the presence of a stereogenic center, all of which bodes well for the successful development of targeted bioactivity. The second is related to fine-tuning the desired activity and pharmacokinetics by selective introduction of fluorine. Historically, both trends were developed separately as innovative and prolific approaches in modern drug design. However, in recent decades, these approaches are clearly converging leading to an ever-increasing number of newly approved pharmaceuticals containing both structural features of amino acids and fluorine.

## 1. Introduction

Pharmaceutical drugs enjoy a tremendously wide range of molecular architectures and chemical functionalities. Nevertheless, the structural analysis allows discerning some general trends guiding the rational design of modern pharmaceuticals. One of these growing trends is the use of amino acid (AA) residues [1,2,3,4,5,6] and related compounds, such as diamines and amino alcohols. AAs represent one of the most ubiquitous classes of naturally occurring compounds serving prolific biological functions [7]. Thus, the inclusion of an AA, or its derivative, in a drug candidate amplifies functional and structural complexity by providing two orthogonal functional amino and carboxyl groups. These aspects usually bode well for the success rate of molecules moving from the discovery phase to the market [8,9,10,11,12]. Another rapidly growing trend in the design of modern drugs is the introduction of fluorine-containing substituents into the structure of a drug candidate [13,14,15,16]. Selective fluorination allows for fine-tuning of the targeted bioactivity and pharmacokinetics. Over the last 20 years, fluorine scanning and editing became rather standard steps in the design of new drugs [17,18,19,20,21].

**Figure 1:**
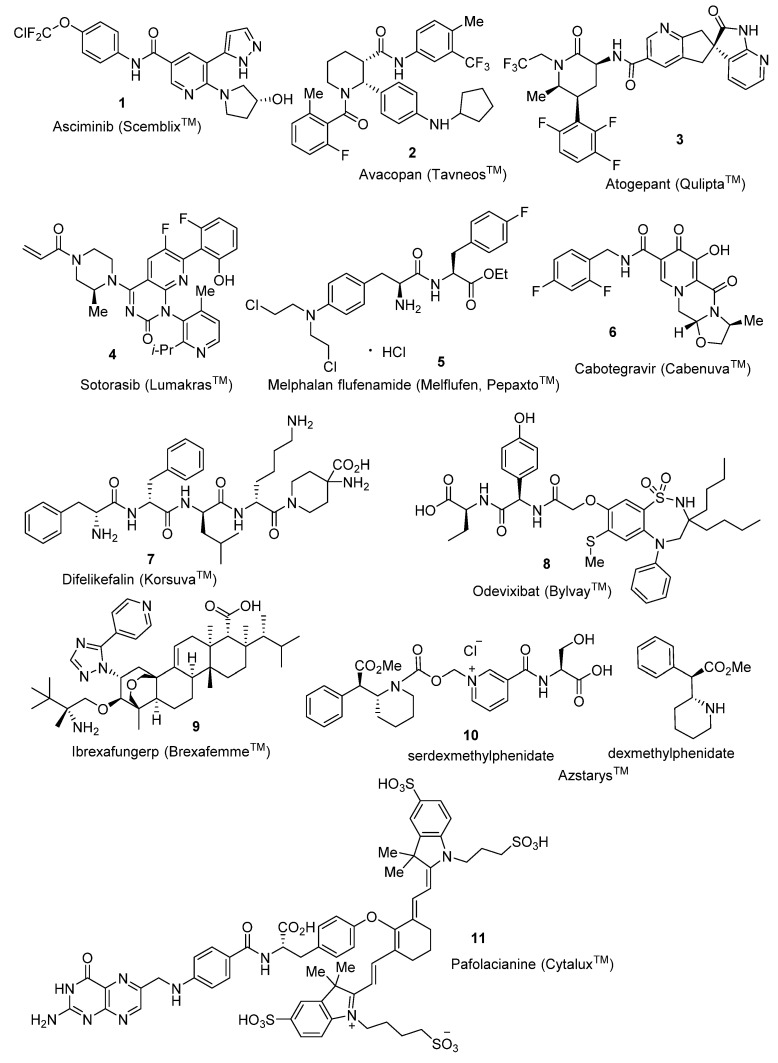
Structures of drugs **1–11**.

In the present review article, we profile the eleven selected drugs approved by the FDA in 2021 (Figure 1), which contain the fragment of amino acid and six of them feature fluorine. In particular, we will discuss the following drugs containing both AA residue and fluorine: Scemblix (**1**), Tavneos (**2**), Qulipta (**3**), Lumakras (**4**), Pepaxto (**5**), and Cabenuva (**6**). The rest of these compounds we are going to profile contain only AA residues, which include Korsuva (**7**), Bylvay (**8**), Brexafemme (**9**), Azstarys (**10**), and Cytalux (**11**). Therapeutic areas of the compounds **1–11** include cancer (**1**,**4**,**5**,**11**), autoimmune disease (**2**), migraine (**3**), HIV (**6**), kidney disease (**7**), skin disease (**8**), vaginal disease (**9**) and attention deficit hyperactivity disorder (**10**). For each of these 11 compounds, we will discuss biological properties and detailed synthesis. In the case of fluorinated molecules (**1–6**), we will also discuss the effect of fluorine introduction and the source of fluorinated materials.

## 2. Asciminib (Scemblix™)

Asciminib **1** is an allosteric inhibitor of the tyrosine kinase activity of the BCR-ABL1 oncoprotein which causes chronic myelogenous leukemia [22]. In contrast to tyrosine kinase inhibitors interacting with the ATP-binding site of BCR-ABL1, asciminib **1** binds to the myristoyl-binding pocket on protein affording high selectivity for inhibition of BCR-ABL1 kinase. Because asciminib **1** does not bind to the ATP-binding site it retains activity against BCR-ABL1 mutations which are associated with acquired resistance toward ATP-competitive drugs [23]. The US FDA approved asciminib **1** as an oral drug for the treatment of adults with Philadelphia chromosome-positive (Ph+) chronic myeloid leukemia and Philadelphia chromosome-positive (Ph+) acute lymphoblastic leukemia in 2021 [24]. This drug may also treat non-malignant diseases associated with abnormally activated ABL1 kinase enzyme [25,26].

**Figure 2:**
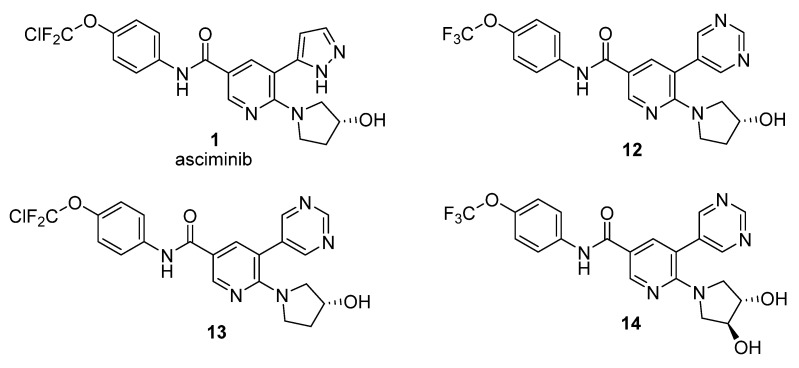
Structure of asciminib **1** and its analogs.

The X-ray crystal structure analysis of compound **12** (Figure 2) bound to the ABL1 protein showed that the CF_3_O-group [13,27] is located in the deepest part of the myristate pocket and one of the fluorine atoms highly interacts with the carbonyl carbon of leucine-359 [28]. Further molecular modeling study indicated that replacing one of the fluorine atoms with chlorine leads to a slightly larger ClCF_2_O-group which can easily be placed in the myristate pocket. This modification improves the activity of compound **13** in biochemical and cellular assays for ABL1 kinase inhibition compared to the CF_3_O-counterpart **12**. Replacing the pyrimidine ring of **13** with a pyrazole ring only slightly reduced the cellular activity of asciminib **1**. However, its hERG activity was markedly reduced. The introduction of an additional hydroxyl group in the pyrrolidine ring of **12** increased the solubility but decreased the cellular activity of **14**.

**Scheme 1:**
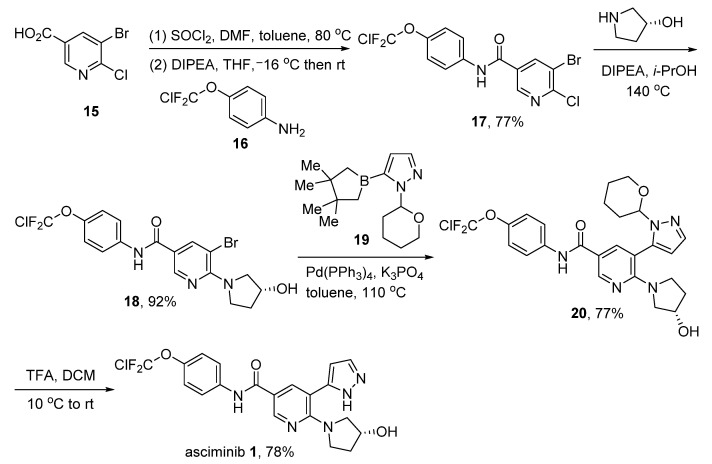
Synthesis of asciminib **1**.

The synthesis of asciminib **1** starts from 5-bromo-6-chloronicotinic acid **15** and 4-(chlorodifluoromethoxy)aniline **16** (Scheme 1) [28]. It should be noted that **16** derivative is commercially available and can be obtained according to the described procedures [29]. The acid chloride derived from acid **15** was condensed with the aniline **16** in the presence of *N*,*N*-diisopropylethylamine (DIPEA) at room temperature to obtain nicotinamide **17** in 77% yield. S_N_Ar displacement of the chlorine atom of **17** by treatment with the enantiomerically pure (*R*)-pyrrolidin-3-ol in the presence of DIPEA at 140 °C afforded the amine **18**. Subsequently, amine **18** was subjected to a Suzuki–Miyaura coupling with tetrahydropyranyl protected (1*H*-pyrazol-5-yl)boronic acid **19** under Pd(PPh_3_)_4_ catalysis at 110 °C providing the protected pyrazole **20** in good yield. Finally, deprotection of tetrahydropyran group with TFA at 10–25 °C gave asciminib **1** in 47% yield over four steps.

## 3. Avacopan (Tavneos™)

Avacopan **2** is an orally bioavailable and highly selective antagonist of human C5aR1 which was developed by ChemoCentryx [30]. The complement anaphylatoxin C5a is a potent inflammatory mediator and exerts its effects by interacting with the C5a receptor 1 (C5aR1) on the surface of neutrophils controlling cell activation and migration [31]. The C5a ligand and its receptor C5aR1 play a central role in the pathogenesis of anti-neutrophil cytoplasmic antibody (ANCA)–associated vasculitis which is characterized by blood vessel inflammation resulting in tissue destruction [32,33]. The US FDA approved avacopan **2** for the treatment of ANCA-associated vasculitis, which is a rare autoimmune disease, in 2021 [30,34].

**Figure 3:**
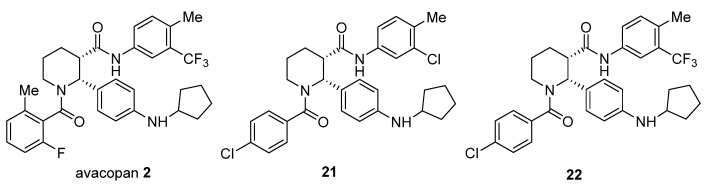
Structures of avacopan **2** and its analogs.

From the structural point of view, avacopan **2** contains fluorine atom and trifluoromethyl group bounded to different aromatic rings which improves C5aR1 inhibition (IC_50_ < 5 nM) compared with chlorinated counterparts **21** and **22** (IC_50_ > 5 nM) (Figure 3) [35,36].

**Scheme 2:**
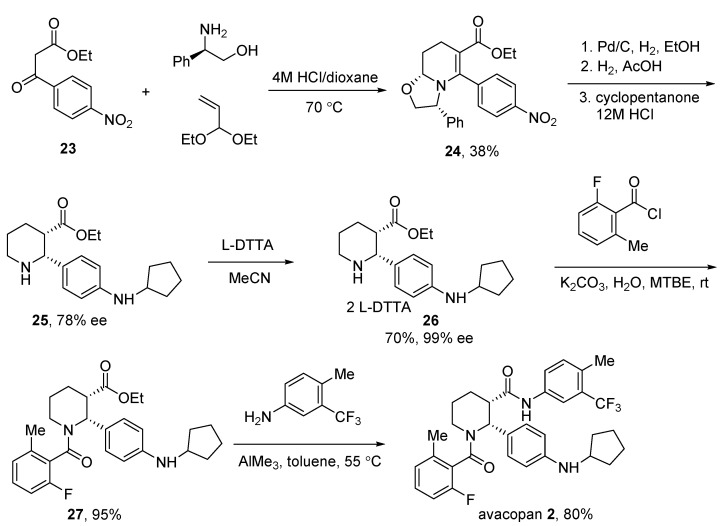
Synthesis of avacopan **2**.

The synthesis of avacopan **2** starts from three-component condensation of ketoester **23**, (*R*)-2-amino-2-phenylethan-1-ol and acrolein diethyl acetal in the presence of catalytic HCl, which gave the bicyclic tetrahydropyridine derivative **24** in 38% yield. Hydrogenation of **24** on 10% Pd/C in ethanol proceeded with good *cis*-diastereoselectivity. The following reduction of the nitro group, reductive removal of the chiral auxiliary and reductive amination of the deprotected piperidine intermediate with cyclopentanone afforded the amino acetate **25** with 78% ee. Chiral resolution [37,38] of **25** by treatment with di-*p*-toluoyl-L-tartaric acid (L-DTTA) in acetonitrile provided di-tartaric salt **26** in 70% yield and 99% ee. Benzoylation of the piperidine nitrogen of **26** with 2-fluoro-6-methylbenzoyl chloride gave amide **27**. Finally, the synthesis of avacopan **2** was conveniently achieved using AlMe_3_-mediated direct coupling of the ester group of **27** and 4-methyl-5-trifluoromethylaniline in good yield (80%) after recrystallization from ethanol/water (Scheme 2).

## 4. Atogepant (Qulipta™)

**Figure 4:**
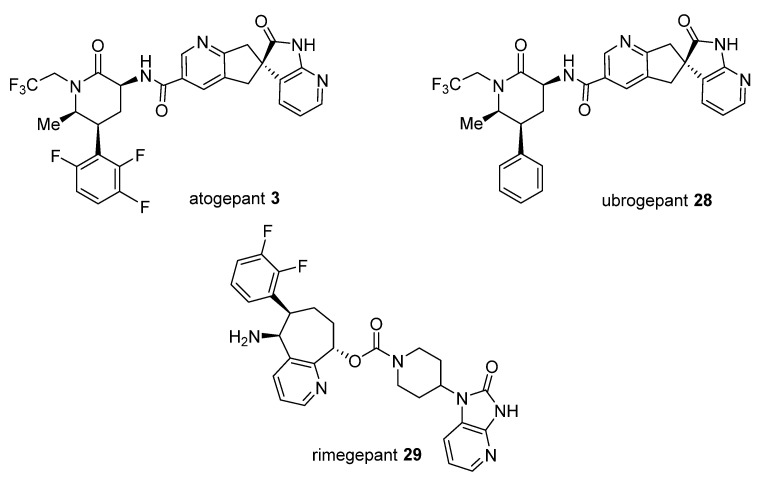
Structures of atogepan **3**, ubrogepant **28**, and rimegepant **29**.

Atogepant **3** is developed by AbbVie as an orally active and selective antagonist of the calcitonin gene-related peptide (CGRP) receptors [39]. CGRP is a neuropeptide widely distributed in the central and peripheral nervous system and regulates cardiac excitability, microvascular permeability, vascular smooth muscle tone, and angiogenesis. CGRP acts by binding to specific cell surface receptors preferentially activating adenylate cyclase [40]. Recently, it was shown that CGRP levels correlate with migraine attacks and CGRP receptors are expressed in the brain regions associated with migraine pathophysiology [41]. Therefore, CGRP receptor antagonists, such as clinical agents atogepant **3**, ubrogepant **28**, and rimegepant **29** (Figure 4), also called gepants, were developed for the treatment of acute migraine headache [42]. The US FDA approved atogepant **3** for the preventive treatment of episodic migraine in adults in 2021 [43].

Atogepant **3** is a polycyclic compound, containing 2-azaspiro[4.4]nonan unit in (*S*) configuration. The spiroazaindane unit is linked with an amide bond to a piperidine-2-one ring bearing 6-(*R*)-methyl and 5-(*R*)-2,3,6-trifluorophenyl substituents. These fluorine substituents provide an increase in atogepant **3** binding affinity for the receptor compared with ubrogepant **28**, which contains an unsubstituted phenyl ring. At the same time, 2,2,2-trifluoroethyl group at the nitrogen atom of piperidine-2-one ring improves the potency and bioavailability of atogepant **3**. While atogepant **3** and ubrogepant **28** contain an aromatic linker, piperidine linker is present in rimegepant **29**.

**Scheme 3:**
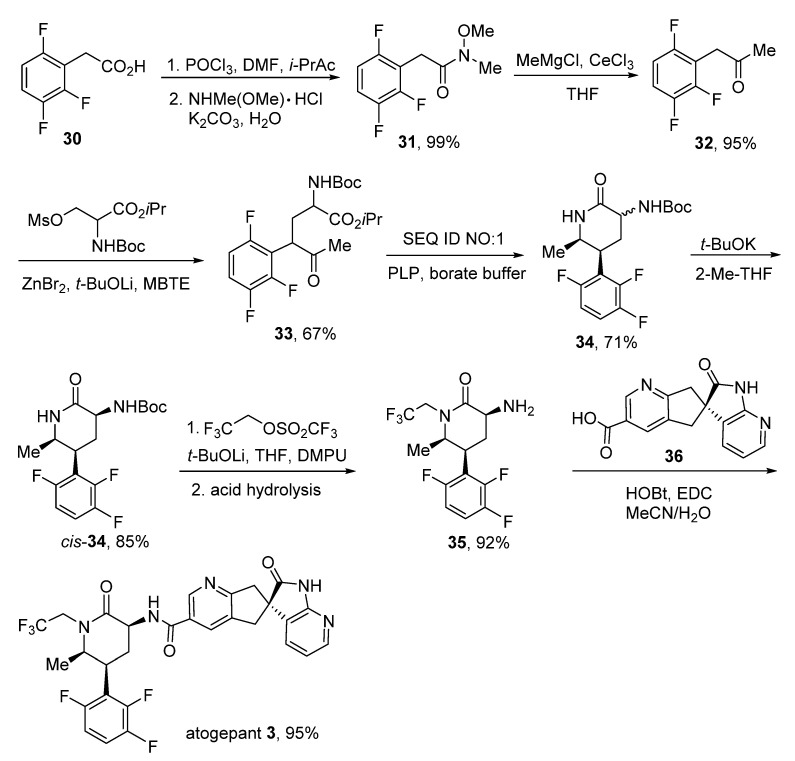
Synthesis of atogepant **3**.

The synthesis of atogepant **3** (Scheme 3) was carried out using fluorinated 2-phenylacetic acid **30** as a starting material [44,45]. Acid **30** was treated with POCl_3_ in isopropyl acetate (*i-*PrAc) at 0 °C and then acid chloride was condensed with *N,O*-dimethylhydroxylamine hydrochloride in the presence of K_2_CO_3_ to yield *N*-methoxy-*N*-methylamide **31** in excellent yield. The conversion of amide **31** into ketone **32** was readily accomplished by treatment with methylmagnesium chloride/cerium(III) chloride in THF. The ketone **32** was alkylated using *N*-(*tert*-butoxycarbonyl)-*O*-(methylsulfonyl)serin isopropyl ester under basic conditions to provide keto ester **33** as one pair of diastereomers. The dynamic kinetic resolution [46,47] of keto ester **33** applying ω-transaminase with pyridoxal 5′-phosphate (PLP) as cofactor provided piperidin-2-one **34** high diastereoselectivity at positions C5 and C6. Treatment of crude *cis*/*trans* mixture **34** with *t-*BuOK in 2-Me-THF provided crystalline *cis*-**34**. *N*-Trifluoroethylation of piperidine-2-one of *cis*-**34** with trifluoroethyl trifluoromethanesulfonate followed by removal of Boc group generated the 3-aminopiperidin-2-one **35** in 92% yield. Coupling of **35** with carboxylic acid **36** in the presence of 1-hydroxybenzotriazole (HOBt) and 1-(3-dimethylaminopropyl)-3-ethylcarbodiimide hydrochloride (EDC) at room temperature gave atogepant **3** in 95% yield as a monohydrate. An alternative method for synthesis of the key 3-aminopiperidin-2-one intermediate **35** including the separation of the racemic mixture of **34** by chiral-HPLC was also described [44].

## 5. Sotorasib (Lumakras™)

Sotorasib **4** is developed by Amgen as a RAS GTPase family inhibitor [48]. Among members of the RAS family of proteins, the KRAS isoform is the most frequently mutated oncogene in cancer and mutations mostly occur in codon 12, such as p.G12D (41%), p.G12V (28%), and p.G12C (14%) [49]. The KRAS protein functions as a signaling molecule capable of subsequent regulation of the proliferation by alternating between a guanosine diphosphate-bound inactive form and a guanosine triphosphate-bound active form. Recently, a series of quinazolinone derivatives were identified as selective covalent inhibitors of KRAS^G12C^ interacting with the cryptic pocket on the surface of the mutated protein [50]. The US FDA approved sotorasib **4** in 2021 for the treatment of adult patients, with KRAS^G12C^-mutated locally advanced or metastatic non-small cell lung cancer (NSCLC) and colorectal cancer, who have received at least one prior systemic therapy [51]. 

**Figure 5:**
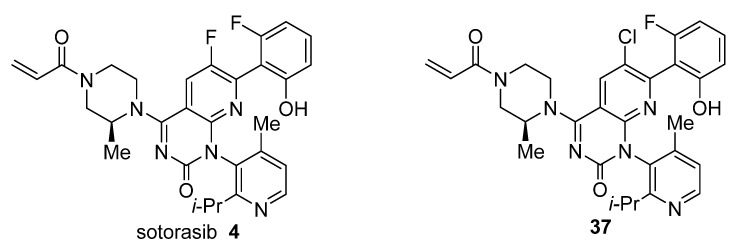
Structure of sotorasib **4** and its analog **37**.

Sotorasib **4** contains a pyrido[2,3-*d*]pyrimidin-2(1*H*)-one unit substituted with 4-methyl-2-(propan-2-yl)pyridin-3-yl, (2*S*)-2-methyl-4-(prop-2-enoyl)piperazin-1-yl, 2-fluoro-6-hydroxyphenyl, and fluoro groups. 2,6-Dialkyl substituents on the pyridine ring constrain biaryls’ rotation around the C-N bond affording the stable atropisomer. Sotorasib **4** contains two fluorine atoms on different aromatic rings and replacing the chloro substituent of compound **37** for fluorine increases bioavailability, although a modest loss of activity in cellular assays is observed (Figure 5) [52].

**Scheme 4:**
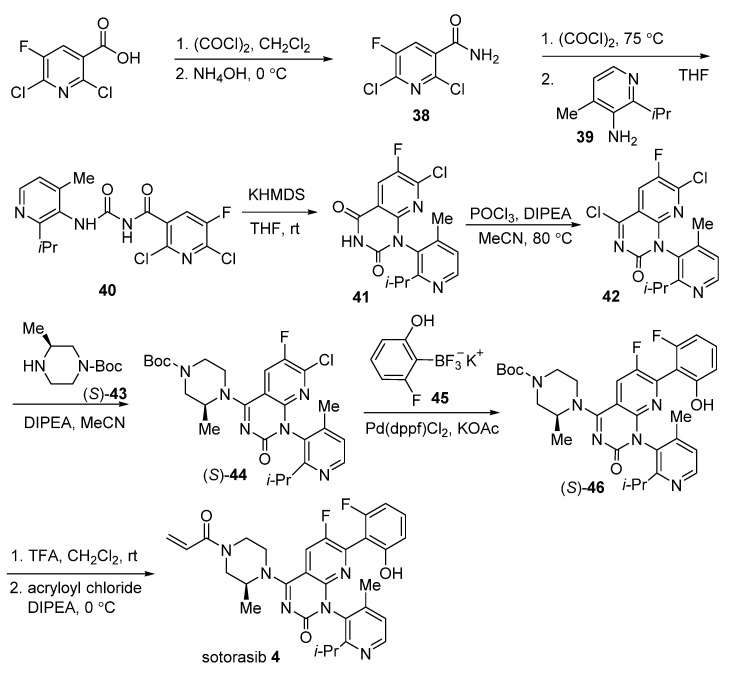
Synthesis of sotorasib **4**.

Multi-step synthesis of sotorasib **4** starts from commercial 2,6-dichloro-5-fluoronicotinic acid (Scheme 4) [53]. The first step consisted of its treatment with oxalyl chloride in CH_2_Cl_2_ providing corresponding acid chloride, which directly reacted with ammonium hydroxide solution in dioxane at 0 °C to give nicotinamide **38**. The reaction of **38** with oxalyl chloride in THF at 75 °C followed by the addition of aminopyridine **39** gave urea **40**. Cyclization of **40** by treatment with potassium hexamethyldisilazide in THF at room temperature afforded **41** after chromatography purification on silica gel. Chlorination of **41** with POCl_3_ in the presence of DIPEA in acetonitrile at 80 °C afforded **42**, which without purification underwent nucleophilic substitution with BOC-protected piperazine **43** to yield **44**. The Suzuki–Miyaura reaction between **44** and potassium trifluoroborate **45** in the presence of PdCl_2_(dppf)^.^CH_2_Cl_2_ complex and potassium acetate at 90 °C gave the coupling product **46**. Finally, sotorasib **4** was obtained by Boc deprotection with TFA; reaction with acryloyl chloride in the presence of DIPEA, and purification by silica gel chromatography.

## 6. Melphalan Flufenamide (Melflufen, Pepaxto™)

**Figure 6:**
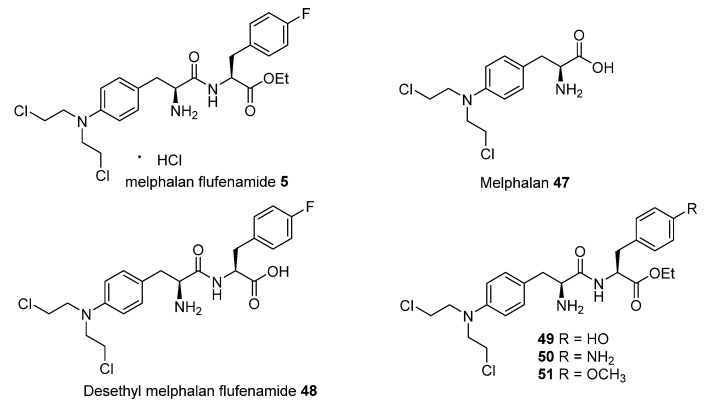
Structures of melphalan flufenamide **5**, its metabolites and analogs.

Melphalan flufenamide **5** is an ethyl ester of a lipophilic dipeptide combining melphalan and L-*p*-fluorophenylalanine and developed by Oncopeptides for the treatment of multiple myeloma and amyloid light-chain amyloidosis. The highly lipophilic melphalan flufenamide penetrates cell membranes and is hydrolyzed in aminopeptidase-positive tumor cells by peptidases and esterases to yield hydrophilic cytotoxic alkylating agents, such as melphalan **47** and desethyl melphalan flufenamide **48** (Figure 6). This results in irreversible DNA damage, apoptosis, and cell death [54]. Melphalan flufenamide **5** shows antitumor activity in multiple myeloma, lymphoma and acute myeloid leukemia cell lines, and primary tumor cells [55]. The application of Melphalan flufenamide **5** enhances efficacy and reduces toxicity compared to melphalan **47**. Structure-activity relationship (SAR) analysis for melphalan-containing dipeptide derivatives **49–51** shows similar cytotoxic activity but lower metabolic stability in comparison with melphalan flufenamide **5** [56]. The US FDA approved melphalan flufenamide **5** in combination with dexamethasone for the treatment of pre-treated adult patients with relapsed or refractory multiple myeloma in 2021 [57].

**Scheme 5:**
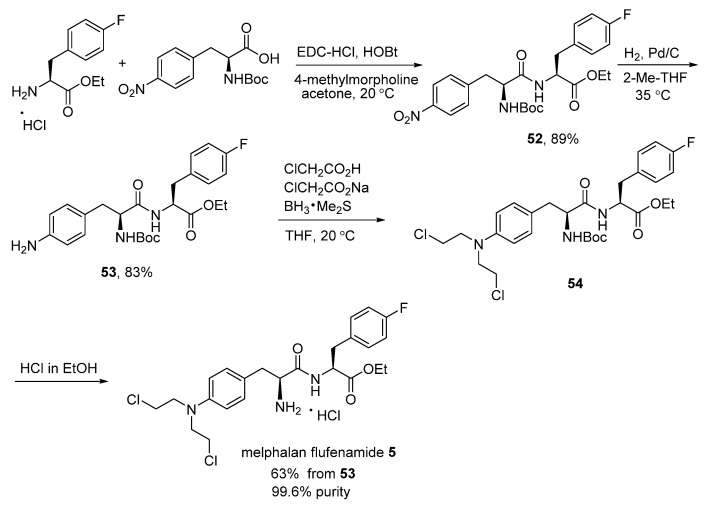
Synthesis of melphalan flufenamide **5**.

A four-step process suitable for commercial production of melphalan flufenamide **5** (Scheme 5) starts from the coupling of 4-fluoro-L-phenylalanine [58] ethyl ester with Boc-4-nitro-L-phenylalanine providing dipeptide **52** in excellent yield (89%) and purity (99.9%) [59,60]. Reduction of the nitro group of **52** with Pd/C as a catalyst in 2-methyltetrahydrofuran afforded **53** in high yield (83%). Next, one step reductive bis-alkylation of amine **53** was achieved using chloroacetic acid, sodium chloroacetate, and the borane-dimethyl sulfide complex as the reducing agent to give *N*,*N*-bis-chloroethylamino derivative **54**. The use of an excess of sodium chloroacetate in the bis-alkylation reaction minimizes side reactions providing a high purity product (99.6%). Thus, *N*,*N*-bis-chloroethylamine functionality can be introduced at the late stage of this process. The deprotection of the *N*-Boc group by treatment of HCl in ethanol at room temperature produced melphalan flufenamide **5** hydrochloride in high purity (99.6%). The overall yield of this four-step process was 47%.

## 7. Cabotegravir (Cabenuva™)

Cabotegravir **6** is developed by ViiV Healthcare as an integrase strand transfer inhibitor (INSTI) of the carbamoyl pyridone type. In the HIV replication cycle, integrase catalyzes the insertion of the viral genome into the genome of host cells. INSTIs prevent the formation of the covalent bond with host DNA by blocking the integrase enzyme, thus inhibiting the incorporation of viral DNA into the host genome [61]. Cabotegravir **6** exhibits slow metabolism, low aqueous solubility, and an extremely long half-life of 21–50 days [62]. Because cabotegravir **6** is metabolized primarily by uridine diphosphate glucuronosyltransferase 1A1, the possibility of interactions with other antiretroviral drugs is minimal [63]. The US FDA approved in 2021 the combination of cabotegravir **6** and non-nucleoside reverse transcriptase inhibitor (NNRTI) rilpivirine as a long-acting injectable regimen for the treatment of HIV-1 in virologically suppressed adults with no suspected resistance to cabotegravir **6** or rilpivirine [64,65].

**Figure 7:**
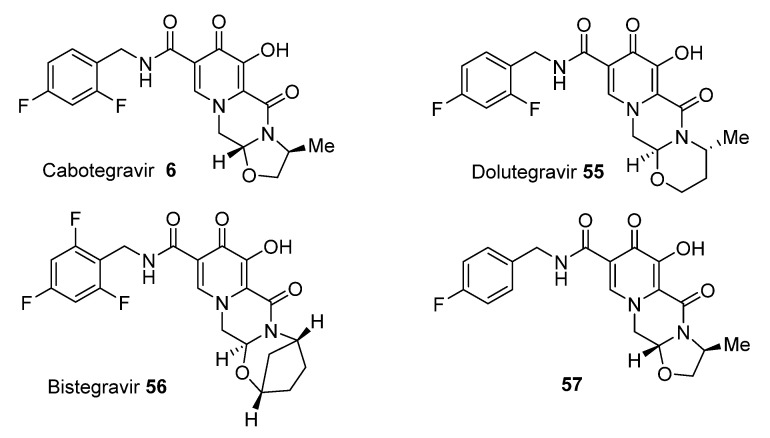
HIV-1 integrase strand transfer inhibitors.

Integrase inhibitors cabotegravir **6**, dolutegravir **55**, and bictegravir **56** are structurally similar compounds with different chiral oxazine ring sizes (Figure 7). In general, the difluorobenzyl derivative cabotegravir **6** showed an improvement in potency against the Q148K compared to its monofluoro analog **57**. The SAR studies showed the importance of the aromatic fluorine atoms in improving the antiviral activity of cabotegravir **6** by approximately four-fold compared with its monofluoro counterpart **57 [66]**.

**Scheme 6:**
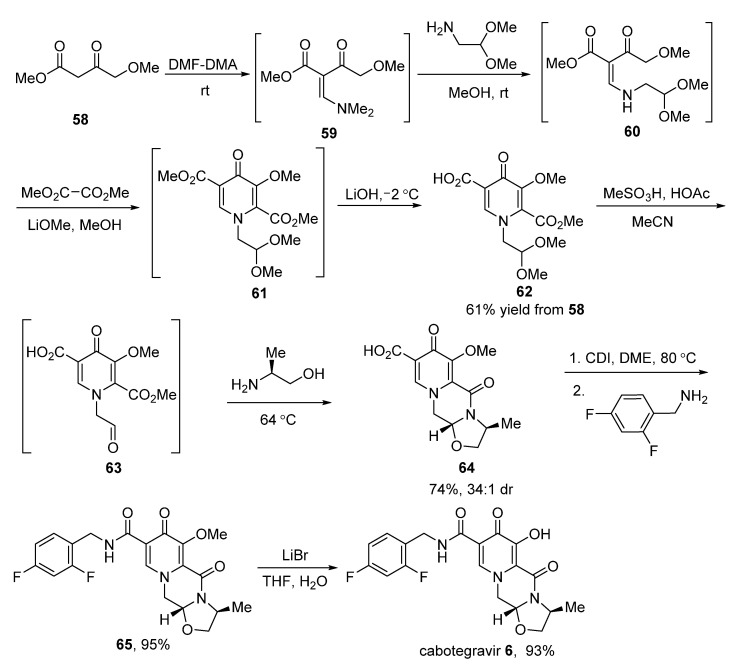
Synthesis of cabotegravir **6**.

Synthetic route to cabotegravir **6** starts from 4-methoxy β-ketoester **58** (Scheme 6) [67,68]. The reaction of ester **58** with neat *N*,*N*-dimethylformamide dimethylacetal (DMF-DMA) at room temperature afforded enamine **59**. After removing excess DMF-DMA, the resulting mixture was treated with aminoacetaldehyde dimethyl acetal and MeOH to afford vinylogous amide **60**. Amide **60** underwent cyclization with dimethyl oxalate in the presence of LiOMe to give pyridinone **61**. Selective hydrolysis of the desired ester group of **61** was effectively achieved with LiOH at low temperature affording the carboxylic acid **62** as a white solid in 61% overall yield from **58**. The acetal deprotection using MeSO_3_H and HOAc in MeCN gave the aldehyde **63** which was directly subjected to the cyclization with (*S*)-alaninol at 64 °C to form the oxazolidine ring in a 34:1 diastereomeric excess. The diastereoselectivity could be improved to 41:1 after recrystallization from methanol. The reaction of carboxylic acid **64** with *N*,*N*′-carbonyldiimidazole (CDI) followed by 2,4-difluorobenzylamine [68] afforded amide **65** in 95% yield. Demethylation was accomplished by using LiBr as a deprotecting agent in THF/water to give the cabotegravir **6** in a 93% yield.

**Scheme 7:**
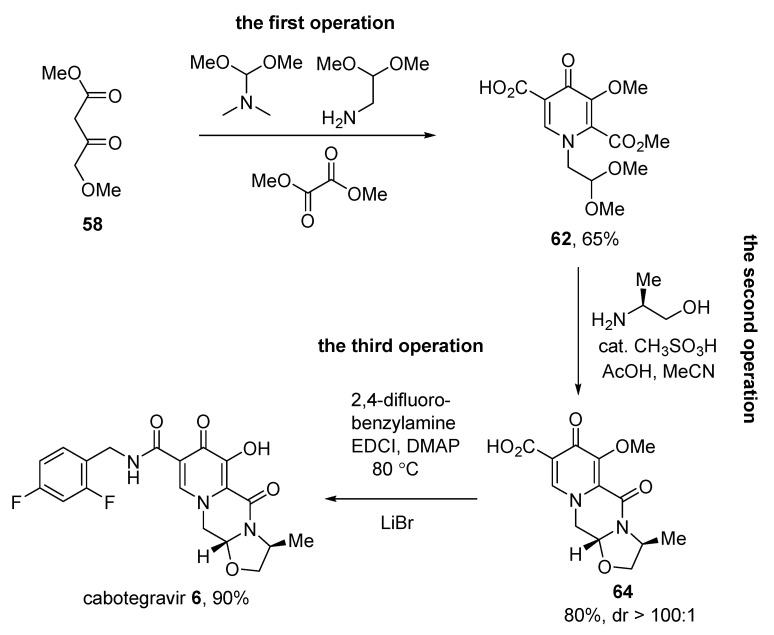
Flow synthesis of the HIV integrase inhibitor cabotegravir **6**.

Recently, a flow system to realize the seven-step synthesis of cabotegravir **6** was developed (Scheme 7) [69]. The flow system consisted of three distinct flow operations and afforded the desired cabotegravir **6** in a better yield compared to the step-by-step procedure. The advantages of the flow system include low cost, time saving, and universality (can be used to obtain antiretroviral pharmaceuticals dolutegravir **55** and bictegravir **56**). This process is scalable and can be used for industrial production.

## 8. Difelikefalin (Korsuva™)

**Figure 8:**
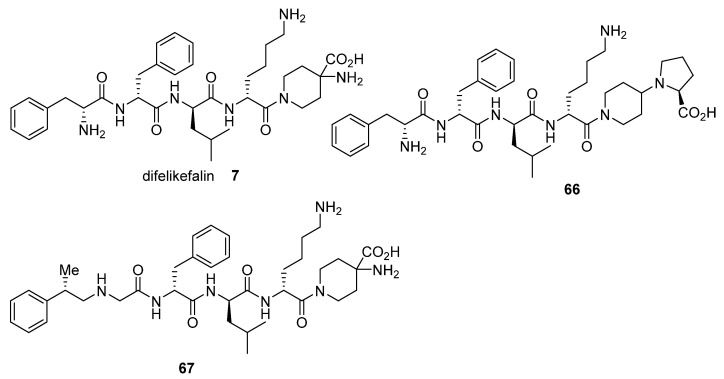
Difelikefalin **7** and its analogs.

Difelikefalin **7**, also called CR845, was developed by Cara Therapeutics as an agonist of the kappa opioid receptor (KOR) for treating the disease of pruritus [70,71]. Difelikefalin **7** is a peptide, and the chemical structure is (D-Phe-D-Phe-D-Leu-D-Lys-[ω(4-aminopiperidine-4-carboxylic acid)]-OH, which contains three natural AAs and one unnatural AA. The SAR studies by Cara Therapeutics disclosed that the unnatural amino acid moiety, 4-aminopiperidine-4-carboxylic acid, was crucial for the activity of the kappa opioid agonist. As shown in Figure 8, replacing the 4-aminopiperidine-4-carboxylic acid with proline led to a decrease in KOR activity, as the KOR EC_50_ value for difelikefalin was 0.048 nM while the value for its analog **66** was 0.075 nM [72]. On the other hand, the other moiety of difelikefalin **7**, the *N*-terminal D-phenylalanine, is also an important pharmacophore for the binding activity of KOR [73]. For example, changing this D-Phe part to another amino acid (**67**) usually led to several folds poorer KOR EC_50_ values. According to the clinical results, difelikefalin **7** received its first and only therapy from the FDA for treating the disease of pruritus associated with chronic kidney disease (CKD-aP) in adults undergoing hemodialysis in August 2021 [74].

**Scheme 8:**
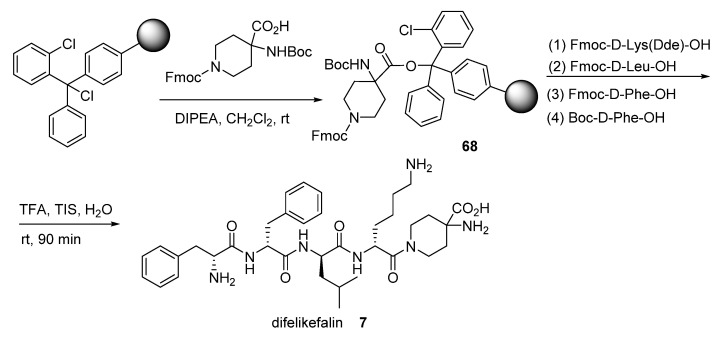
Synthesis of difelikefalin **7**.

Since difelikefalin **7** is a peptide, Cara Therapeutics developed a solid phase peptide synthesis method for its preparation [72]. As presented in Scheme 8, the peptide synthesis started from 2-chlorotrityl chloride resin, which was coupled with *N*-Boc-amino-(4-*N*-Fmoc-piperidinyl) carboxylic acid in the presence of DIPEA in dichloromethane at room temperature [75]. The resulting resin **68** was then coupled with Fmoc-D-Lys(Dde)-OH, Fmoc-D-Leu-OH, Fmoc-D-Phe-OH, and Boc-D-Phe-OH, which was followed by the removal of the protecting groups and resin in the presence of TFA and triisopropylsilane (TIS) at room temperature for 90 min. The obtained crude difelikefalin **7** was precipitated from the ether and purified on a HPLC column.

## 9. Odevixibat (Bylvay™)

**Figure 9:**
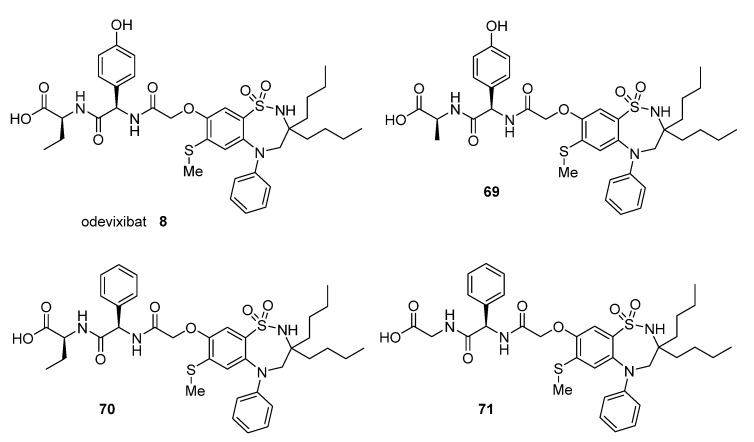
Structures of odevixibat **8** and analogs.

Odevixibat **8** was developed by Albireo Pharma, Inc. as a potent and selective inhibitor of the ileal bile acid transporter (IBAT) for the treatment of all subtypes of progressive familial intrahepatic cholestasis (PFIC) [76,77,78]. Odevixibat **8** was demonstrated to be safe and its oral administration was well tolerated [79]. Odevixibat **8** is a chiral compound, which contains one 2-aminobutyric acid and one 4-hydroxyphenylglycine amino acid moieties, and one 2,3,4,5-tetrahydrobenzo[*f*][1,2,5]thiadiazepine 1,1-dioxide heterocyclic unit. The SAR studies by Albireo disclosed that odevixibat **8** exhibits better IBAT inhibitory activity compared with its analogs. The dipeptide moiety is very important for the activity [80]. For example, the IC_50_ (IBAT) value for odevixibat **8** was 0.16 nmol/L, which was two-fold over its analog **69** (Figure 9) containing an alanine unit instead of a 2-aminobutanoate species (IC_50_ = 0.3 nM). Additionally, changing the 4-hydroxyphenylglycine AA moiety to phenylglycine (**70**) led to slightly decreased activity, i.e., an IC_50_ value of 0.18 nM. In particular, if both of the two AAs of odevixibat **8** were changed into others (**71**), a dramatically lower activity was observed with an IC_50_ value of 1.2 nM (about eight-fold). Odevixibat **8** obtained its first approval from the EU and FDA in July 2021 for the treatment of PFIC with more than six-month of age in the EU and the treatment of pruritus in all subtypes of PFIC in the USA [81].

**Scheme 9:**
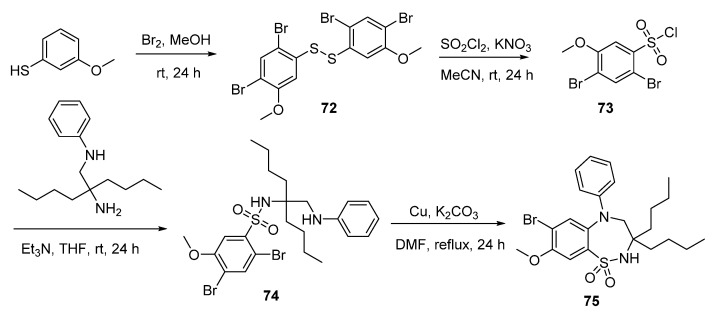
Synthesis of the intermediate **75**.

Astrazeneca patented a synthetic method for the preparation of odevixibat **8**, which employed poly-substituted 2,3,4,5-tetrahydrobenzo[*f*][1,2,5]thiadiazepine 1,1-dioxide **75** as the key intermediate [82]. The synthesis of intermediate **75** was presented in Scheme 9 with 3-methoxybenzenethiol as the starting material [83]. Treatment of 3-methoxybenzenethiol with bromine in methanol at room temperature for 24 h afforded 1,2-bis(2,4-dibromo-5-methoxyphenyl)disulfide (**72**), which was converted into sulfonyl choride **73** via the reaction with sulfuryl chloride in the presence of potassium nitrate in acetonitrile. Then, the reaction of sulfonyl chloride **73** with 2-butyl-*N*^1^-phenylhexane-1,2-diamine at room temperature generated the sulfonamide intermediate **74** with triethylamine as a base. Finally, the Cu-promoted intramolecular cyclization reaction of sulfonamide **74** under reflux in DMF for 24 h provided the key 2,3,4,5-tetrahydrobenzo[*f*][1,2,5]thiadiazepine 1,1-dioxide intermediate **75**.

**Scheme 10:**
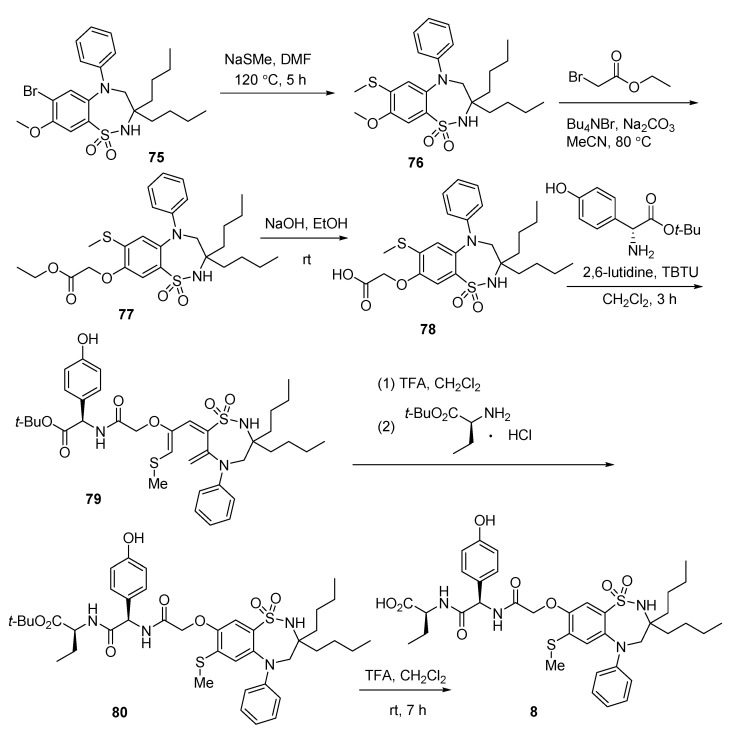
Synthesis of odevixibat **8**.

After the key heterocyclic moiety was constructed, the bromo group on the phenyl ring of the intermediate **75** was substituted with methylthio group via reaction with NaSMe in DMF at 120 °C for 5 h, generating the intermediate **76** (Scheme 10) [82]. Subsequently, the intermediate **76** was subjected to the reaction with ethyl 2-bromoacetate and sodium carbonate in the presence of Bu_4_NBr yielding the ester **77**. Hydrolysis of ester **77** with the use of sodium hydroxide provided the acid **78**, which underwent the condensation reaction with *tert*-butyl (*R*)-2-amino-2-(4-hydroxyphenyl)acetate in the presence of *O*-(benzotriazol-1-yl)-*N*,*N*,*N*′,*N*′-tetramethyluronium tetrafluoroborate (TBTU) and 2,6-lutidine in dichloromethane. The generated *tert*-butyl ester **79** was treated by TFA in dichloromethane followed by the second peptide condensation reaction with *tert*-butyl (*S*)-2-aminobutanoate, providing the dipeptide **80**. Finally, the target odevixibat **8** was obtained via the hydrolysis of the intermediate **80** under acidic conditions.

## 10. Ibrexafungerp (Brexafemme™)

**Figure 10:**
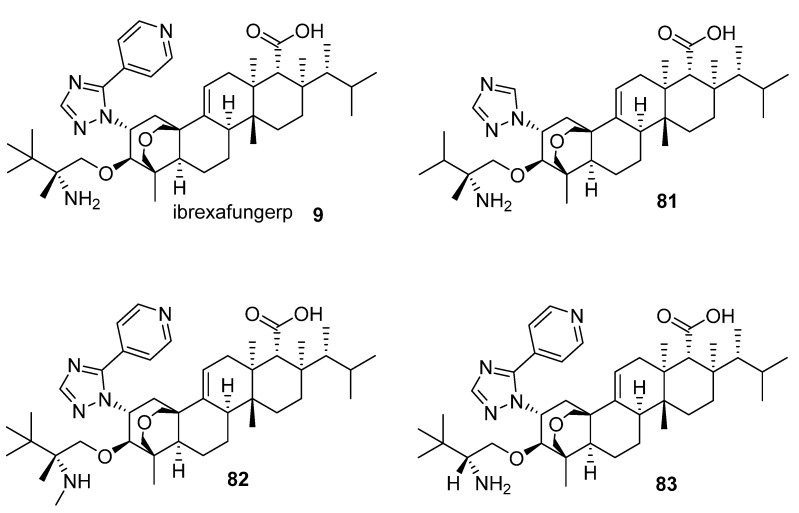
Structures of ibrexafungerp **9** and analogs.

Ibrexafungerp **9**, with the trade name Brexafemme™, was approved in June of 2021 by the FDA for the treatment of vulvovaginal candidiasis (VVC). As a derivative of the natural product enfumafungin, it was developed by the company Scynexis [84]. Ibrexafungerp is a triterpenoid antifungal drug for adult and post-menarchal pediatric females with VVC. Although the specific binding site of ibrexafungerp is not clear, it could blockade the biosynthesis of β-1,3-glucan, a crucial component of the fungal cell wall [85]. Ibrexafungerp **9** is a chiral compound derived from the natural product enfumafungin containing a pyridyl triazole moiety and an aminoether moiety, which are suggested to be quite important for the drug efficiency. For example, the IC_50_ of ibrexafungerp for β-1,3-glucan synthase inhibition in Candida albicans (MY 1055) microsomal membrane fragments is 0.001 μg/mL. When the pyridyl group on triazole unit is replaced by hydrogen, **81** (Figure 10) demonstrates a raised IC_50_ value of 0.007 μg/mL. Additionally, when the substituents on the aminoether units change slightly, the IC_50_ values of **82** and **83** are 0.002 and 0.007 μg/mL, respectively [86]. Actually, ibrexafungerp **9** (ED_99_ = 0.84 mpk vs. *C. albicans*) was developed via SAR optimization by the Scynexis Inc. of the substituent on triazole moiety from their previously reported β-1,3-glucan synthesis inhibitor, MK-5204 (ED_99_ = 6.4 mpk vs. *C. albicans*) [86]. Ibrexafungerp is supplied as tablets for oral administration. It should be mentioned that this non-azole antifungal drug is not suitable for pregnant patients based on animal toxicity studies [84].

**Scheme 11:**
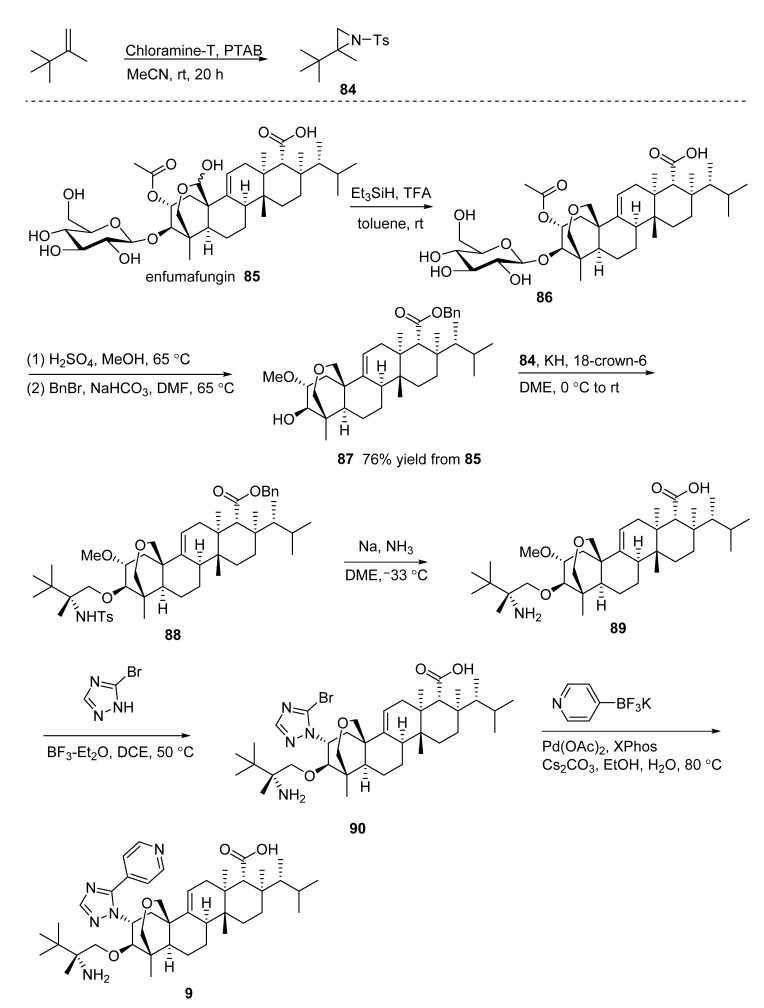
Synthesis of ibrexafungerp **9**.

The synthetic method for the preparation of ibrexafungerp **9** by Merck and Scynexis Inc. is presented in Scheme 11, which used a natural product, enfumafungin (**85**) as the starting material [86]. First, aziridination of 2,3,3-trimethylbut-1-ene with the use of chloramine-T and phenyltrimethylammonium tribromide (PTAB) in acetonitrile at room temperature provided the aziridine intermediate **84** [87]. On the other hand, the reduction of the bridging hemiacetal of enfumafungin (**85**) by triethylsilane in the presence of TFA afforded the deoxyenfumafungin intermediate **86**. Then, the removal of the glycoside by sulfuric acid in methanol and the replacement of the acetoxy group by methyl ether followed by the esterification of the carboxylic acid group via treatment with benzyl bromide and sodium bicarbonate afforded the intermediate **87** [87]. Subsequently, intermediate **87** was subjected to an alkylation reaction with aziridine intermediate **84** in the presence of KH and 18-crown-6 resulting in the amine **88**, which then underwent a Birch reduction reaction to remove the tosyl and benzyl protecting groups. The obtained free aminoether **89** was treated with 3-bromo-1,2,4-triazole in the presence of borontrifluoride diethyletherate in dichloroethane providing the intermediate **90** [88]. Finally, the Suzuki coupling reaction between the intermediate **90** and potassium pyridine-4-trifluoroborate gave the targeted ibrexafungerp **9**.

## 11. Serdexmethylphenidate and Dexmethylphenidate (Azstarys™)

Serdexmethylphenidate **10** is a prodrug of d-methylphenidate, which is an ammonium salt containing two AA moieties. As shown in Figure 1, the two AAs, methyl (*R*)-2-phenyl-2-((*R*)-piperidin-2-yl)acetate and L-serine were linked by nicotinoyl and carbamate. Serdexmethylphenidate and d-methylphenidate capsule (SDX/d-MPH) has been developed as a new stimulant and used in children with attention deficit hyperactivity disorder (ADHD). The randomized, double-blind, dose-optimized studies showed that a significant treatment effect for SDX/d-MPH compared with placebo was observed from 1 to 10 h postdose [89,90,91]. In March 2021, SDX/d-MPH developed by Corium was approved by the FDA with the trade name Azstarys™ for attention deficit hyperactivity disorder (ADHD) in patients 6 years of age and older.

**Scheme 12:**
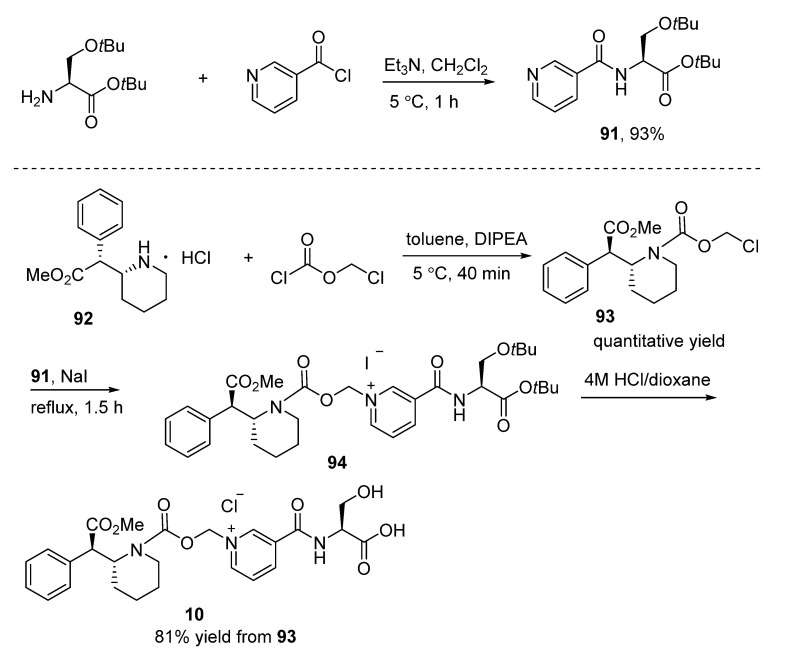
Synthesis of serdexmethylphenidate **10**.

Kempharm, INC. patented a method for the preparation of serdexmethylphenidate **10** in 2018, which used *tert*-butyl *O*-(tert-butyl)-L-serinate (H-Ser(*t*Bu)-O*t*Bu) as the starting material (Scheme 12) [92]. First, the addition of nicotinoyl chloride into the solution of H-Ser(*t*Bu)-O*t*Bu in dichloromethane in the presence of triethylamine at 5 °C afforded the nicotinoyl-Ser(*t*Bu)-O*t*Bu **91** in 93% yield after 1 h. On the other hand, the reaction of methyl (*R*)-2-phenyl-2-((*R*)-piperidin-2-yl)acetate hydrochloride [93] (**92**) with chloromethyl chloroformate using DIPEA as a base generated the amide **93** in quantitative yield, which then was refluxed with nicotinoyl-Ser(*t*Bu)-O*t*Bu **91** and NaI in acetone resulting in the iodide salt intermediate **94**. Finally, the iodide salt intermediate **94** was treated with 4M HCl in dioxane, affording the target serdexmethylphenidate **10**. It should be mentioned that an anion exchange resin, Dowex 1*8 chloride was used to replace the anionic counter iodide ion with the chloride ion in the last step.

## 12. Pafolacianine (Cytalux™)

**Figure 11:**
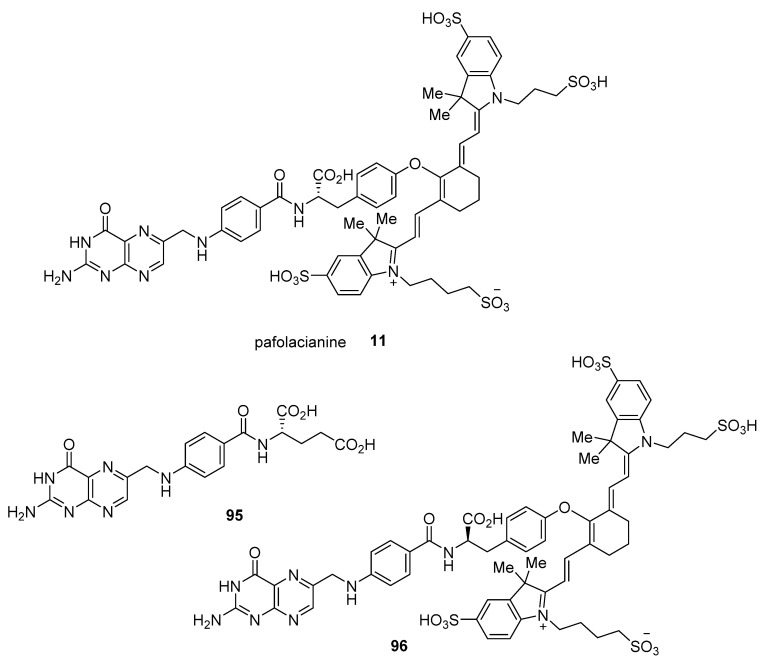
Pafolacianine **11** and its analogs.

Pafolacianine **11**, also named OTL38, was a tumor-targeted near-infrared fluorescent dye, which provides visual evidence of its remarkable cancer-illuminating capabilities. It was developed by On Target Laboratories as an adjunct to visual inspection and palpation by providing intra-operative imaging of folate receptor positive (FR+) ovarian cancer according to its fundamental properties [94]. Pafolacianine **11** contains a pteroyl amide unit, a tyrosine AA moiety and a S0456 fluorescent species. The evaluation of the binding affinity was performed, and the results disclose that L-tyrosine is important for the binding affinity. The relative affinity (RA) value calculated for folic acid **95** (Figure 11) and pafolacianine **11**, was 1.0 and 0.8, respectively. However, when changing the L-tyrosine into D-tryrosine, the RA value for compound **96** will be 0.09 [95]. Based on the safety and efficacy results of phase III [96], pafolacianine sodium injection obtained its approval from the FDA in November 2021 with the trade name Cytalux™ for use to help identify ovarian cancer lesions [97,98].

**Scheme 13:**
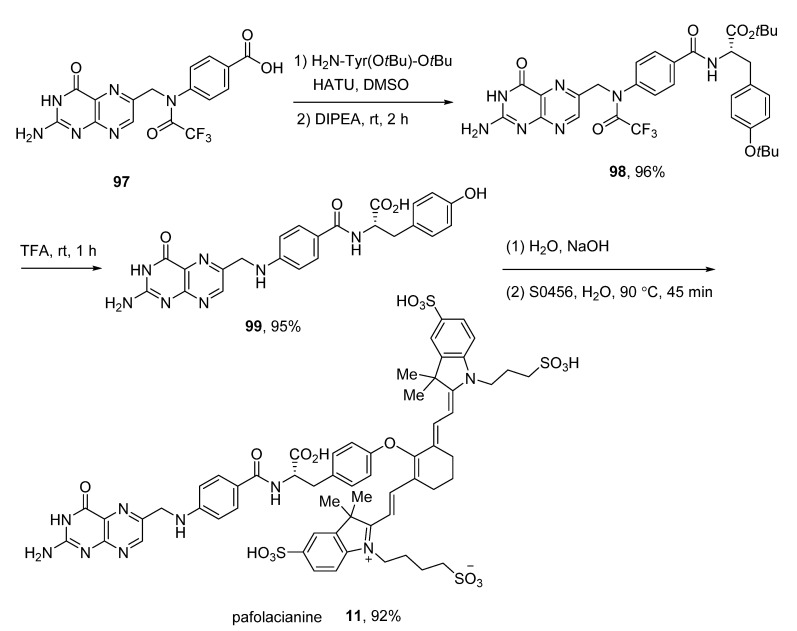
Synthesis of pafolacianine **11**.

The synthesis of pafolacianine **11** is shown in Scheme 13, which used commercially available N^10^-trifluoroacetylpteroic acid **97** as the starting material [94,95]. First, condensation of **97** with NH_2_-L-Tyr(O*t*Bu)-O*t*Bu with the use of 2-(7-azabenzotriazol-1-yl)-*N*,*N*,*N*′,*N*′-tetramethyluronium hexafluorophosphate (HATU) as the condensing reagent in the presence of DIPEA afforded the corresponding pteroyl-N^10^(TFA)-Tyr(O*t*Bu)-O*t*Bu intermediate **98** in 96% yield at room temperature after 2 h. Then, the removal of the *tert*-butyl protecting groups via treatment with TFA at room temperature generated the pteroyl-N^10^(TFA)-Tyr(OH)-CO_2_H intermediate **99** in 95% yield. Subsequently, the intermediate **99** was treated with aqueous NaOH solution and then reacted with S0456 at 90 °C for 45 min providing the target pafolacianine **11** in 92% yield.

## 13. Conclusions

The growing acceptance of peptides (biopharmaceuticals) and peptidomimetics (small-molecule pharmaceuticals) as drugs clearly indicates that AA derivatives will remain the preferred structural platform in the development of modern pharmaceuticals. Some of the problematic issues associated with AAs application include racemization, proteolytic and microsomal metabolism, clearance rates, and the membrane permeability of AA-derived compounds. These issues can be addressed by the strategic substitution of fluorine for hydrogen and the incorporation of trifluoromethyl and chlorodifluoromethyl groups. Steric, electronic and physical properties of the fluorinated groups [99,100,101,102] can be rationally applied to enhance configurational stability, reduce proteolytic and microsomal degradation, slow down clearance rates and enhance membrane permeability [103,104,105,106,107], allowing to ameliorate the inherent problems associated with AA-derivatives. Of special note is that promiscuity and off-target toxicity of drug candidates are shown to be reduced by the introduction of elements of chirality, in particular, stereogenic centers. Quite unsurprisingly, all 11 drugs reviewed in this work are chiral, containing at least one and up to 12 stereogenic carbons. In this regard, it should be emphasized that the reported stereochemical outcome of the enantioselective reactions discussed in this review is missing the issue of the SDE properties [108,109] of enantiomerically enriched intermediates and final products. It has been unambiguously demonstrated that fluorine [110,111,112], chiral amines [113,114], α- [115,116], and β-amino acids [117] are SDE-phoric groups, showing a significant magnitude of SDE under a variety of physicochemical transformations. As a result of the SDE ignorance, the reported data of stereochemical outcome can be collected with substantial mistakes leading to an erroneous presentation of the synthetic value and mechanistic interpretation of the corresponding reactions [118,119]. Studying the SDE properties of chiral molecules is an important issue in laboratory practice and drug development, reflecting the current standards in the administration of chiral drugs as pure enantiomers [120,121,122].

## Data Availability

Not applicable.
